# Burying our mistakes: Dealing with prognostic uncertainty after severe brain injury

**DOI:** 10.1111/bioe.12737

**Published:** 2020-03-02

**Authors:** Mackenzie Graham

**Affiliations:** ^1^ Uehiro Centre for Practical Ethics Oxford University, UK and Wellcome Centre for Ethics and Humanities, Oxford University UK

**Keywords:** brain injury, end‐of‐life treatment, decision‐making, neuroethics, prognostic uncertainty

## Abstract

Prognosis after severe brain injury is highly uncertain, and decisions to withhold or withdraw life‐sustaining treatment are often made prematurely. These decisions are often driven by a desire to avoid a situation where the patient becomes ‘trapped’ in a condition they would find unacceptable. However, this means that a proportion of patients who would have gone on to make a good recovery, are allowed to die. I propose a shift in practice towards the routine provision of aggressive care, even in cases where the probability of survival and acceptable recovery is thought to be low. In conjunction with this shift, I argue in favour of a presumption towards withdrawing life‐sustaining treatment, including artificial nutrition and hydration, when it becomes clear that a patient will not recover to a level that would be acceptable to them. I then respond to three potential objections to this proposal.

## 1 INTRODUCTION

Prognosis after severe brain injury, particularly in the first few hours or days, is highly uncertain. A patient may have a small chance of recovering to complete or near‐complete independence, remain permanently unconscious, or end up somewhere between these two extremes, with varying degrees of probability. Increased certainty about outcome emerges as the patient recovers (or their condition worsens), but it may be weeks or even months before physicians can provide any specific and accurate statements about expected long‐term recovery.

Despite this uncertainty, up to 50% of decisions to withhold or withdraw treatment after severe brain injury occur within 72 hours of admission to hospital.1
Turgeon, A. F., Lauzier, F., Simard, J. F., Scales, D. C., Burns, K E., Moore, L. … Fergusson, D. A. (2011). Mortality associated with withdrawal of life‐sustaining therapy for patients with severe traumatic brain injury: A Canadian multicentre cohort study. *Canadian Medical Association Journal*, *183*(14), 1581–1588.10.1503/cmaj.101786PMC318507421876014
 In many cases, this is too early to accurately predict patient outcome. As a result, a proportion of patients that would have made a good or even complete recovery if they had received treatment are allowed to die.

Early decisions to withhold or withdraw life‐sustaining treatment reflect the perceived need to make decisions quickly regarding continuing care, before life‐sustaining treatment becomes unnecessary. After a severe brain injury, there is a limited period of time during which the patient’s death can be quickly brought about by withholding or withdrawing life‐sustaining treatment (e.g., mechanical ventilation). Physicians and surrogate decision‐makers are faced with a dilemma. Providing treatment allows for the patient to make a good recovery, but also risks them ending up in a state they would find unacceptable, with no means of ending their lives. Withholding treatment ensures that the patient will not be trapped in a state of unacceptable disability, but removes any chance of a favourable outcome.

The significant proportion of patients that have treatment withdrawn or withheld within the first 72 hours suggests that in many cases, this dilemma is resolved by allowing some patients to die prematurely, rather than risking an unacceptable recovery. I think this strategy is mistaken. I propose a shift in practice towards the routine provision of aggressive care, even in cases where the probability of survival and acceptable recovery is thought to be low. When prognosis is uncertain, and the prior wishes of the patient are unclear, it is appropriate to provide aggressive treatment. In conjunction with this shift, I argue in favour of a presumption towards withdrawing life‐sustaining treatment, including artificial nutrition and hydration, when it becomes clear that a patient will not recover to a level that would be acceptable to them.

Adopting this treatment strategy is preferable to current practice for several reasons. First, it gives the patient the best possible chance of recovery and allows for the possibility that a physician’s initial assessment may be incorrect. Treatment is withdrawn only after it has proven to be ineffective, and not withheld on the assumption that it will be ineffective. Second, it mitigates one of the primary factors against the provision of aggressive treatment in the early stages after brain injury, namely, that it may result in patients being ‘trapped’ in a state they would find unacceptable. Third, it allows surrogate decision‐makers to delay making an irreversible decision (allowing the patient to die) until prognosis is more certain, and allows for a change in course if it is concluded that continued treatment will not lead to a desirable outcome. Fourth, it prevents patients who will not recover, and who are no longer benefitting from life‐sustaining treatment, from languishing in long‐term care.

## 2 TREATMENT AFTER SEVERE BRAIN INJURY

Emergency care after severe brain injury focuses on ensuring adequate oxygen, maintaining blood pressure, and preventing further injury to the brain. In many cases, however, a patient will not receive all available means of treatment. Several studies have demonstrated a strong correlation between decisions to limit therapy, and the physician’s prediction that the probability of patient survival is low (e.g., less than 10%).2
Cook, D., Rocker, G., Marshall, J., Sjokvist, P., Dodek, P., Griffith, L. … Level of Care Study Investigators and the Canadian Critical Care Trials Group. (2003). Withdrawal of mechanical ventilation in anticipation of death in the intensive care unit. *New England Journal of Medicine*, *349*, 1123–1132; White, D. B., Curtis, J. R., Lo, B., & Luce, J. M. (2006). Decision to limit life‐sustaining treatment for critically ill patients who lack both decision‐making capacity and surrogate decision‐makers. *Critical Care Medicine*, *34*(8), 2053–2059.10.1097/01.CCM.0000227654.38708.C116763515
 Indeed, when a patient is expected to have a poor outcome, they may not receive aspects of intensive care—including intubation or ventilation, osmotic diuretics, intra‐cranial pressure monitoring, and neurosurgery—that would be provided to patients with a more positive predicted outcome.

As many as 70% of deaths following acute brain injury are the result of withholding or withdrawing life sustaining treatment.3
Turgeon, A. F., Lauzier, F., Burns, K. E., Meade, M. O., Scales, D. C., Zarychanski, R., … Canadian Critical Care Trials Group. (2013). Determination of neurologic prognosis and clinical decision making in adult patients with severe traumatic brain injury: a survey of Canadian intensivists, neurosurgeons, and neurologists. *Critical Care Medicine*, *41*(4), 1086–1093.10.1097/CCM.0b013e318275d04623385104
 However, it has been suggested that physician recommendations to withhold or withdraw life‐sustaining treatment within the first 72 hours of hospital admission may often be premature.4
Ibid.; Harvey, D., Butler, J., Groves, J., Manara, A., Menon, D., Thomas, E., & Wilson, M. (2018). Management of perceived devastating brain injury after hospital admission: a consensus statement from stakeholder professional organizations. *British Journal of Anaesthesia*, *120*(1), 138–145.10.1016/j.bja.2017.10.00229397121
 For example, Chamoun and colleagues conducted a retrospective review of 189 patients presenting with a Glasgow Coma Score (GCS) of 3 at hospital admission due to severe traumatic brain injury.5
Chamoun, R. B., Robertson, C. S., & Gopinath, S. P. (2009). Outcome in patients with blunt head trauma and a Glasgow Coma Scale score of 3 at presentation. *Journal of Neurosurgery*, *111*(4), 683–687.10.3171/2009.2.JNS08817PMC279806019326973
 The reported mortality rate in patients with a GCS of 3 is very high, approaching 100% when associated with bilateral fixed and dilated pupils. Yet this study found that at six month follow‐up, 13% of all patients had achieved a good functional outcome (i.e., a Glasgow Outcome Score of 4 or 5, meaning moderate disability and good recovery, respectively. Moderate disability indicates independence, but unable to resume work, school, or all previous social activities. Good recovery indicates minor neurological or psychological deficits, but able to resume normal daily life).

Similarly, Elmer and colleagues estimate that 26% of patients who have life‐sustaining treatment withdrawn within 72 hours after brain injury would have survived, with 64% of these potential survivors going on to make a good recovery (i.e., score of 3 or less on modified Rankin scale. A score of 3 indicates moderate disability, meaning the person requires some help, but can walk without assistance. A score of 2 indicates slight disability, meaning the person is unable to perform all previous activities, but able to look after own affairs without assistance. A score of 1 indicates no disability, despite some symptoms, while a score of 0 indicates no symptoms.)6
Elmer, J., Torres, C., Aufderheide, T. P., Austin, M. A., Callaway, C. W., Golan, E., … Zive, D. M. (2016). Association of early withdrawal of life‐sustaining therapy for perceived neurological prognosis with mortality after cardiac arrest. *Resuscitation*, *102*, 127–135.10.1016/j.resuscitation.2016.01.016PMC483423326836944
 This would amount to approximately 2,300 people per year in the United States being withdrawn from care when they would have survived, nearly 1,500 of which would have had a favourable recovery (at worst, moderate disability).

## 3 PROGNOSTIC UNCERTAINTY

A major reason for uncertainty relating to recovery from severe brain injury is the poor discriminatory quality of prognostic markers. Several factors have been associated with prognosis after severe brain injury, including patient age, sex, cause of injury, Glasgow Coma Score, pupil reactivity, and results of computed tomography. Specific factors, such as haematoma volume greater than 60ml, hydrocephalus, and intraventricular haemorrhage, are well‐accepted predictors of poor outcome after intracerebral haemorrhage, but some patients with several of these features may survive with only moderate degrees of disability.7
Smith, M. (2012). Treatment withdrawal and acute brain injury: An integral part of care. *Anaesthesia*, *67*(9), 941–945.10.1111/j.1365-2044.2012.07304.x22861501



Scoring systems based on these factors have been developed as an early prognostic measure for patients with severe brain injury, but have proven unsatisfactory in the prognosis of individual patients. This issue is exacerbated when prognostic models developed in one context are applied to a different clinical environment without appropriate recalibration,8
Harvey, op. cit*.* note 4.
 or when they do not reflect the therapeutic possibilities afforded by current technology or care practices. For example, the common use of prehospital sedation has diminished the predictive power of the Glasgow Coma Scale.9
Balestreri, M., Czosnyka, M., Chatfield, D. A., Steiner, L.A., Schmidt, E.A., Smielewski, P., … Pickard, J. D. (2004). Predictive value of Glasgow Coma Scale after brain trauma: change in trend over the past ten years. *Journal of Neurology, Neurosurgery and Psychiatry*, *75*(1), 161–162.PMC175744114707332



So‐called ‘self‐fulfilling prophecies’ can also reinforce the perceived validity of prognostic schemes. For example, the bilateral absence of somatosensory evoked potentials (SSEPs) has been suggested as a reliable indicator of poor outcome after severe brain injury. However, in many studies measuring the predictive value of SSEPs, patients may be withdrawn from life‐sustaining treatment. Thus, it is unclear if the correlation between bilateral absence of SSEPs and poor outcome is the result of an underlying neurophysiological cause, or whether the withdrawal of life‐sustaining treatment is influencing the observed correlation. Moreover, clinicians may be influenced by the results of these studies to recommend withholding or withdrawing care when SSEPs are bilaterally absent, further reinforcing this association.10
Izzy, S., Compton, R., Caradang, R., Hall, W., & Muehlschlegel, S. (2013). Self‐fulfilling prophecies through withdrawal of care: Do they exist in traumatic brain injury, too? *Neurocritical Care*, *19*(3), 347–363.10.1007/s12028-013-9925-z24132565



## 4 THE ROLE OF PHYSICIAN VALUES

Decisions to withhold or withdraw life‐sustaining treatment may also be complicated by disagreement between physicians and surrogate decision‐makers about whether continued treatment is beneficial for the patient. In many cases, clinician recommendations that life‐sustaining treatment should be withheld or withdrawn are strongly influenced by the clinician’s own beliefs, values, and practice style. One of the strongest predictors of withholding or withdrawing life‐sustaining treatment is the physician’s view that severe disability is likely to result from continued care. Yet research suggests that physicians tend to be overly pessimistic when assessing prognosis.11
Moore, N. A., Brennan, P. M., & Baille, J. K. (2013). Wide variation and systematic bias in expert clinicians’ perceptions of prognosis following brain injury. *British Journal of Neurosurgery*, *3*, 340–343.10.3109/02688697.2012.75440223461749



Individual physicians may operate according to different beliefs about the ‘sanctity of life’, or how they interpret poor outcomes (i.e., what makes a ‘life worth living’), and these values can colour how they communicate potential treatment decisions to the family.12
Turgeon, op. cit*.* note 3.
 This can lead to considerable differences in practice, with one study demonstrating that the proportion of patients being withdrawn from life‐sustaining treatment within three days of admittance to the intensive care unit varied between 30.4% and 92.9% across six level‐one trauma centres in Canada.13
Turgeon, op. cit*.* note 1.
 Similarly, a survey by Turgeon and colleagues asked 455 intensivists, neurosurgeons, and neurologists to evaluate the prognosis at one year of a hypothetical patient. Approximately one third agreed or strongly agreed that the patient would have an unfavourable outcome, one third disagreed or strongly disagreed that the patient’s prognosis was unfavourable, and the remaining third were neutral. Yet 80% of respondents said they would be uncomfortable or very uncomfortable recommending the withdrawal of care in this situation.14
Ibid.



## 5 DECIDING UNDER UNCERTAINTY

The lack of reliable prognostic data, combined with the potential for physician bias, means that decisions to withhold or withdraw life‐sustaining treatment within the first 72 hours after severe brain injury are highly uncertain. Both physicians and decision‐makers may be uncertain of the exact probabilities for various outcomes, as well as how the patient themselves would have evaluated these outcomes. Surrogate decision‐makers may also be uncertain of how to balance the physician’s prognosis, and their own beliefs about the patient’s values or wishes.15
Boyd, E. A., Lo, B., Evans, L. R., Malvar, G., Apatira, L., Luce, J. M., & White, D. B. (2010). ‘It’s not just what the doctor tells me’: Factors that influence surrogate decision‐makers’ perceptions of prognosis. *Critical Care Medicine*, *38*(5), 1270–1275.10.1097/CCM.0b013e3181d8a217PMC353083820228686



One potential strategy here is to simply wait until prognosis becomes clearer. For example, the Joint Professional Standards committee and the Neurocritical Care Society have recommended an observation period of up to 72 hours when withholding or withdrawing life‐sustaining treatment is being considered.16
Souter, M. J., Blissitt, P. A., Blosser, S., Bonomo, J., Greer, D., Jichici, D. … Yeager, S. (2015). Recommendations for the critical care management of devastating brain injury: Prognostication, psychosocial, and ethical management: A position statement for healthcare professionals from the Neurocritical Care Society. *Neurocritical Care*, *23*(1), 4–13.10.1007/s12028-015-0137-625894452
 If the patient’s condition continues to deteriorate during the period of observation, the clinical team may consider this an appropriate trigger for discussions with the surrogate decision‐maker about withdrawal of life‐sustaining treatment. Conversely, if the patient shows evidence of recovery, treatment decisions can be reconsidered.

## 6 THE ‘WINDOW OF OPPORTUNITY’

The problem with adopting a policy of delay is that the range and likelihood of potential outcomes may be influenced by when treatment decisions are made. Immediately after severe brain injury, there is a period—sometimes called ‘the window of opportunity’—during which a patient is physiologically unstable and withholding life‐sustaining treatment is likely to result in death. Delaying decisions to withhold or withdraw life‐sustaining treatment risks foreclosing the option of allowing the patient to die of their injuries. Surrogates and physicians must then wait for another life‐threatening condition to occur (e.g., pneumonia, infection), which can go untreated in order to bring about the patient’s death. Depending on the condition, this can result in a great deal more suffering for the patient, and their family.

Surrogate decision‐makers are thus faced with the following choice: (1) risk an unknown probability of a bad outcome (i.e., severe disability or prolonged dying) for an unknown probability of a good outcome (i.e., good functional recovery); or (2) accept an almost certain probability of a different bad outcome (i.e., death), and forego a good outcome.

The prevalence of early withdrawal of life‐sustaining treatment suggests a tendency towards the second option amongst many decision‐makers. In order to avoid the worst possible outcome, surrogate decision‐makers must give up the possibility of a good outcome. I propose altering the conditions of these options by changing our treatment practice.

## 7 AGGRESSIVE TREATMENT

I argue that the most appropriate course of action for surrogate decision‐makers and clinicians in light of uncertainty is to provide aggressive treatment to patients in the immediate stages after injury. When prognosis is uncertain, and the patient’s wishes are unknown, the presumption should be to treat.

### 7.1 Early aggressive treatment improves outcome

Aggressive treatment in the early stages after brain injury maximizes the probability of an acceptable recovery for the patient. The US Brain Trauma Foundation17
Carney, N., Totten, A. M., O'Rielly, C., Ullman, J. S., Hawryluk, G. W. J., Bell, M. J., † Ghajar, J. (2017). Guidelines for the management of severe traumatic brain injury, fourth edition. *Neurosurgery*, *80*(1), 6–15.10.1227/NEU.000000000000143227654000
 has issued a set of treatment recommendations for the management of patients with severe traumatic brain injury. These include certain types of decompressive craniectomy, drainage of cerebralspinal fluid to lower intracranial pressure, barbiturate therapy, and invasive intracranial pressure monitoring in all patients with an abnormal CT scan.

Numerous studies have demonstrated that early and aggressive interventions—including invasive treatments like decompressive craniectomy and intracranial pressure monitoring—are associated with a decrease in mortality and an increase in favourable outcomes after severe brain injury.18
Whitemore, R. G., Thawani, J. P., Grady, M. S., Levine, J. M., Sanborn, M. R., & Stein, S. C. (2012). Is aggressive treatment of traumatic brain injury cost effective? *Journal of Neurosurgery*, *116*(5), 1106–1113; Stein, S. C., Georgoff, P., Meghan, S., Mirza, K. L., & El Falaky, O. M. (2010). Relationship of aggressive monitoring and treatment to improved outcomes in severe traumatic brain injury. *Journal of Neurosurgery*, *112*(5), 1105–1112; Chieregato, A., Venditto, A., Russo, E., Martino, C., & Bini, G. (2017). Aggressive medical management of acute traumatic subdural hematomas before emergency craniotomy in patients presenting with bilateral unreactive pupils. A cohort study. *Acta Neurochirurgica*, *159*(8), 1553–1559.10.1007/s00701-017-3190-428435989
 Importantly, timing of surgical intervention is closely related to patient outcome, with multiple studies demonstrating that patients who underwent surgery within four hours of their injury had a statistically significant decrease in mortality, compared to those who delayed surgery.19
Feinberg, M., Mai, J. C., & Ecklund, J. (2015). Neurosurgical management in traumatic brain injury. *Seminars in Neurology*, *35*(1), 50–56.10.1055/s-0035-154424425714867



### 7.2 Early aggressive treatment increases certainty

A presumption in favour of early aggressive treatment acknowledges the uncertainty of prognosis after severe brain injury. The decision to provide or withhold life‐sustaining treatment is based on a prediction that the patient will recover to a level that is acceptable to them. The more evidence that physicians and surrogate decision‐makers have in support of one outcome over another, the more likely their decision will promote the best interests of the patient. Providing aggressive care early on provides more opportunity to observe and assess the patient, and more time for consciousness to re‐emerge. It allows time to establish whether the patient is trending upwards towards recovery, trending downwards, or remaining stagnant. This information is what is needed to inform a decision to continue or withdraw life‐sustaining treatment.

The level of certainty about the patient’s outcome that is needed to justify a decision to withhold or withdraw life‐sustaining treatment will depend on a number of factors, including the prior wishes of the patient (if these are known), and the views of the patient’s family. Unfortunately, there is no diagnostic test that can confirm that a disorder of consciousness, such as a coma or vegetative state, is permanent. According to the Multi‐Society Task Force on Persistent Vegetative State, the chance of recovery in adult patients in a vegetative state for less than three months after a severe head injury is 33%, from three to six months was 13%, and from six to 12 months is 6%.20
Multi Society Task‐Force on PVS (1994). Medical aspects of the persistent vegetative state. *NEJM*, *330*, 1499–1508.10.1056/NEJM1994052633021077818633



These guidelines acknowledge that a diagnosis of permanent vegetative state is a prediction that awareness will never recover, but that this cannot be known with certainty. Patients may be misdiagnosed, or their condition may change over time. In fact, recent research has demonstrated that as many as 19% of patients repeatedly diagnosed as being in a vegetative state can demonstrate awareness through functional neuroimaging.21
Fernandez‐Espejo, D., & Owen, A. M. (2013). Detecting awareness after severe brain injury. *Nature Reviews Neuroscience*, *14*, 801–809.10.1038/nrn360824088810



It is impossible to specify an appropriate level of certainty to justify a decision to withhold or withdraw life‐sustaining treatment that would apply in all cases. However, in most cases, 72 hours is insufficient. At the same time, it may be possible to achieve a level of certainty sufficient to justify withdrawing life‐sustaining treatment well before a disorder of consciousness would be considered ‘permanent’. Arguing in favour of a strict time limit for treatment belies the heterogeneous nature of severe brain injury, and the unique needs and concerns of patients and families. What is required is a careful approach to withholding and withdrawing life‐sustaining treatment early after brain injury that appreciates the uncertainty inherent in these decisions.

## 8 WITHDRAWAL OF TREATMENT

When it is clear that a patient will not make a recovery that is acceptable to them, or that they would not want to continue living in their current state, they should be removed from life‐sustaining treatment and be allowed to die. If the patient remains on life‐sustaining treatment like mechanical ventilation or artificial nutrition or hydration, these interventions can be ethically withdrawn, on the grounds that the patient (via their surrogate‐decision‐maker) has the right to refuse any treatment intervention they do not (or would not) want. This right is grounded in the patient’s right to self‐determination and bodily integrity.

It has been argued that artificial nutrition and hydration is not like other forms of life‐sustaining treatment (e.g., mechanical ventilation or haemodialysis), and as such, decisions to withhold or withdraw it should be treated differently. Proponents of this view often claim that providing artificial nutrition and hydration is not a medical intervention, but a form of basic care—like relief from pain—that should not be denied to anyone. They may also argue that causing a patient’s death through starvation or dehydration is cruel and inhumane.

The decision to withhold or withdraw artificial nutrition and hydration should be treated the same as any other decision to forego treatment. A patient—or a surrogate decision‐maker acting on their behalf—has the right to refuse any treatment; whether the intervention is considered ‘natural’ or ‘artificial’ is not morally significant. Indeed, the provision of artificial nutrition and hydration is a highly invasive intervention, and can lead to considerable discomfort, infection, and other serious complications for the patient.

Moreover, the belief that withdrawing artificial nutrition and hydration is cruel or painful is not supported by empirical evidence. As Cochrane and Truog describe, ‘observations of patients who have refused artificial nutrition and hydration have consistently shown that they die peacefully and without suffering … Far from being a painful way to die, this mode of death appears to be a tolerable and natural form of the dying process.’22
Truog, R. D., & Cochrane, T. I. (2005). Refusal of hydration and nutrition: Irrelevance of the ‘artificial’ vs ‘natural’ distinction. *Archives of Internal Medicine*, *165*(22), 2574–2576.10.1001/archinte.165.22.257416344412
 Patients who display signs of suffering as a result of this process can be provided with sedatives and analgesics. Other measures, such as providing the patient with ice chips to alleviate the discomfort of a dry mouth, can also be provided.

Decisions to withhold or withdraw artificial nutrition and hydration should be based on an assessment of the potential harms and benefits to the patient of continued treatment. The appropriate question is not whether it is in the best interests of the patient for artificial nutrition and hydration to be withdrawn, but whether it is in the best interests of the patient for artificial nutrition and hydration to continue. In the early stages after severe brain injury, the potential benefits of continued treatment, including artificial nutrition and hydration, are considerable, because the patient still has an uncertain—and potentially substantial—chance of recovery. However, when there is greater certainty about the prognosis of the patient, and an acceptable recovery is less likely, justification must be given for continuing to provide artificial nutrition and hydration.

For example, if a patient is in a permanent vegetative state, continued treatment is no longer in their best interests. These patients are incapable of experience of any kind, and continued life cannot be a benefit to them. Conversely, patients diagnosed as vegetative with covert awareness may possess sufficient cognitive capacities to make their lives worth living.23
Graham, M. (2017). A fate worse than death? The well‐being of patients diagnosed as vegetative with covert awareness. *Ethical Theory and Moral Practice*, *20*, 1005–1020.
 These patients may be capable of enjoyment, as well as suffering, and provided with the right care, they may be able to achieve an acceptable level of well‐being. For these patients, continued life could be in their best interests, and justify the continued provision of artificial nutrition and hydration. Of course, some patients might find this kind of life unacceptable, and in these cases, withdrawing life‐sustaining treatment would be justified on the grounds that they were not benefitting from continued treatment.

Evaluating a patient’s quality of life, and specifically when a patient’s life is no longer worth living, is highly challenging. It requires surrogates to consider the patient’s previously expressed wishes and preferences, as well as the values, motivations, desires, and other subjective interests they held prior to their injury. Surrogates must also account for the fact that these values and interests might have changed post‐injury. Indeed, studies suggest that surrogates often perform poorly in judging whether a patient would want to continue treatment.24
Shalowitz, D. I., Garrett‐Mayer, E., & Wendler, D. (2006). The accuracy of surrogate decision‐makers: A systematic review. *Archives of Internal Medicine*, *166*(5), 493–497.10.1001/archinte.166.5.49316534034
 There may also be disagreement amongst family members about whether a patient would consider their life worth living. Nevertheless, treatment decisions must be made, including decisions to withhold or withdraw life‐sustaining treatment. When a patient is no longer benefitting from life‐sustaining treatment, it should be removed.

Allowing surrogate decision‐makers to refuse life‐sustaining interventions on behalf of the patient, when doing so is determined to be in their best interests, effectively removes the constraints on surrogate decision‐making caused by the window of opportunity. If allowing the patient to die continues to be an option open to surrogate decision‐makers, even after the patient is no longer dependent on life‐sustaining treatment, surrogate decision‐makers can pursue treatment without having to worry that the patient may become ‘trapped’ in a state they would find unacceptable. They can pursue the best outcome for the patient, without having to avoid what they may consider to be the worst possible outcome.

One might infer from this argument that if it is permissible to withdraw artificial nutrition and hydration from a patient, it would also be permissible (or even preferable) to provide active euthanasia. Indeed, both appear to be cases of intentional killing. However, I argue that artificial nutrition and hydration should only be provided when doing so is of benefit to the patient. If the patient no longer has a life worth living, it follows that they are no longer benefitting from the provision of this treatment. Because it is permissible for a patient to refuse any treatment (including artificial nutrition and hydration), a surrogate can refuse it on the patient’s behalf. A right to active euthanasia cannot be similarly grounded in a right to refuse treatment, though it could be justified in other ways. However, my purpose in this paper is not to provide an argument in favour of or against the moral permissibility of active euthanasia.

## 9 OBJECTIONS

### 9.1 Harms of aggressive treatment

One potential objection to the kind of treatment strategy I describe is that even if we minimize the possibility of an unacceptable outcome for the patient, aggressive treatment might still lead to significant harms. Aggressive treatment is highly invasive, and patients may suffer during the period of recovery. It may also take several days or weeks before we can be sufficiently confident in the prognosis of the patient to warrant a decision to withdraw treatment, and the patient may be suffering during this time.

Some degree of suffering on the part of the patient after severe brain injury may be unavoidable, regardless of whether a patient is withdrawn from care early after their injury. But this suffering can be justified by the benefit of a more certain prognosis. Suppose a patient has a 10% chance of making a good recovery upon admission to hospital. Under normal circumstances, this patient would not be offered aggressive treatment, and would likely die. However, suppose that providing this patient with aggressive treatment would increase their chances of a good recovery from 10% to 25%. Whether the patient’s suffering is justified depends not on whether the patient ultimately recovers, but whether the 15% increase in probability of recovery is worth the suffering the patient experiences. This will depend on the disvalue the patient would assign to the suffering, and the value that they would assign to their recovery. Judicious use of medication can help to ameliorate the physical suffering resulting from aggressive treatment.

Nevertheless, in some cases the increase in probability of good recovery resulting from aggressive treatment may not offset the additional suffering of the patient caused by aggressive treatment. For some patients, no amount of increase in the probability of recovery would be worth the suffering of existing in a state of severe disability, even for a short time prior to the withdrawal of life‐sustaining treatment. All that can be done in this situation is for surrogate decision‐makers to use their best judgement regarding the values of the patient. If surrogate decision‐makers believe that aggressive treatment would not be in the best interests of the patient, they should refuse it on the patient’s behalf.

### 9.2 Withholding treatment vs. withdrawing treatment

A second objection pertains to the putative difference between withholding and withdrawing life‐sustaining treatment. A presumption towards aggressive treatment will lead to a greater number of patients being provided with life‐sustaining treatment, and thus, a potentially greater number of patients who will need to be withdrawn from life‐sustaining treatment (rather than simply having had treatment withheld). However, there is disagreement over whether withdrawing life‐sustaining treatment once it has been started, and simply withholding life‐sustaining treatment, are equally permissible.

On the one hand, the view that there is no moral difference between withholding and withdrawing treatment is widely articulated in ethics guidelines in both the United States and the United Kingdom (AMA, 2016; General Medical Council. 2006).25
American Medical Association (2016). *AMA Code of medical ethics opinions on caring for patients at the end of life.* Available at: https://www.ama-assn.org/delivering-care/ethics/withholding-or-withdrawing-life-sustaining-treatment; General Medical Council (2010). Treatment and care towards the end of life: Good practice in decision‐making.
 This view is justified by consideration of a patient’s best interests. If a treatment is not in a patient’s best interests, a physician is clearly justified in withholding it, and would be equally justified in withdrawing it for the same reasons. Conversely, if a treatment is in a patient’s best interests, it would be equally wrong for a physician to withhold it as it would be to begin treatment and then withdraw.

On the other hand, the widespread agreement amongst philosophers and legal scholars about the moral equivalence of withholding and withdrawing life‐sustaining treatment has done little to influence the views of clinicians. In fact, many clinicians do not agree that withholding and withdrawing treatment are morally equivalent.26
Sprung, C. L., Paruk, F., Kissoon, N., Hartog, C. S., Lipman, J., Du, B., … Feldman, C. 2014. The Durban World Congress Ethics Round Table Conference Report: I. Differences between withholding and withdrawing life‐sustaining treatments. *Journal of Critical Care*, *29*, 890–895; Chung, G. S., Yoon, J. D., Rasinski, K. A., & Curlin, F. A. (2016). US physicians’ opinions about distinctions between withdrawing and withholding life‐sustaining treatment. *Journal of Religion and Health*, *55*(5), 1596–1606.



Physicians may have a variety of reasons for drawing this distinction. They may be uncertain about what is permitted by law or other professional standards.27
Carlet, J., Thijs, L. G., Antonelli, M., Cassell, J., Cox, P., Hill, N. … Thompson, B. T. (2004). Challenges in end‐of‐life care in the ICU. *Intensive Care Medicine*, *30*, 770–784.10.1007/s00134-004-2241-515098087
 They may be psychologically uncomfortable with actively stopping a life‐prolonging intervention, as well as the public nature of the act, particularly when this pertains to withdrawing artificial nutrition and hydration.28
Chung, op. cit*.* note 26.
 They may feel more directly responsible for patient death, given that patients die much more frequently and quickly after withdrawal, than after withholding treatment (98% compared to 68% after 72 hours).29
Sprung, C. L., Cohen, S.L., Sjokvist, P., Baras, M., Bulow, H. H., Hovilehto, S. … Woodcock, T. (2003). End‐of‐life practices in European intensive care units: the Ethicus study. *JAMA*, *290*, 790–797.10.1001/jama.290.6.79012915432
 They may also want to avoid potentially difficult discussions with families about reasons for withdrawing treatment, discussions which can be avoided if treatment is simply withheld.30
Sprung, op. cit*.* note 26.
 Conversely, some physicians may feel that they have an obligation to relieve some of the decision‐making burden of surrogates, by strongly recommending that treatment be withheld.31
Wilson, M.E., Rhudy, L. M., Ballinger, B. A., Tescher, A. N., Pickering, B. W., & Gajic, O. (2013). Factors that contribute to physician variability in decisions to limit life support in the ICU: A qualitative study. *Intensive Care Medicine*, *39*(6), 1009–1018.10.1007/s00134-013-2896-x23559079



Cultural practices can also influence decisions to withhold or withdraw life‐sustaining treatment. In the United States, Canada, and most countries in northern Europe (e.g., the United Kingdom, the Netherlands, France, and Belgium), the withdrawal of life‐sustaining treatment—including artificial nutrition and hydration—is much more common than in southern European countries (e.g., Spain, Portugal, Greece, and Italy), or Asian countries like Japan, China, and South Korea.32
Levin, P. D., & Sprung, C. L. (2003). Cultural differences at the end of life. *Critical Care Medicine*, *31*(5), S354–S357; Vincent, J. L. (2001). Cultural differences in end of life care. *Critical Care Medicine*, *29*(2), S52–S55..10.1097/01.CCM.0000065275.30220.D212771582
 Moreover, in North America, where patient autonomy is highly valued, family involvement on the patient’s behalf in end‐of‐life decision‐making is quite common, whereas physicians play a much larger role in decision‐making in many European countries. In contrast, decisions about life‐sustaining treatment in China and Japan are often made entirely by the patient’s family (even to the exclusion of the patient).33
Ibid.



My argument does not rest on the moral equivalence of withholding and withdrawing treatment. It requires only that withholding and withdrawing treatment are both morally permissible in cases where continued treatment is no longer beneficial to the patient. If a treatment is no longer of clinical benefit to the patient it should not be provided, whether or not it has already commenced. Similarly, a potentially beneficial treatment should not be withheld in order to avoid the challenges of potentially having to withdraw treatment later. For example, a controversial approach to this problem is the use of ‘no escalation of therapy’ orders, in which a patient will continue to receive their current therapy, but will not receive new ones.34
Thompson, D. R. (2014). ‘No escalation of treatment’ as a routine strategy for decision‐making in the ICU: pro. *Intensive Care Medicine*, *40*(9), 1372–1373; Curtis, J. R., & Rubenfeld, G. D. (2014). ‘No escalation of treatment’ as a routine strategy for decision‐making in the ICU: con. *Intensive Care Medicine*, *40*(9), 1374–1376.
 This practice has been defended as a ‘middle‐ground’ for families that will not accept the withdrawal of life‐sustaining treatment. However, continuing to provide ineffective treatment to the patient for the sake of the surrogates or families, is not typically in a patient’s best interest. It does not satisfy the goal of returning the patient to an acceptable quality of life, nor of ensuring comfort in dying.

Nevertheless, the psychological difference for the patient’s family between withholding and withdrawing treatment should not be ignored. Families may experience more stress or guilt about the prospect of actively withdrawing treatment from a patient in the chronic stage of injury, compared to withholding treatment in the early stages after injury. They may be misinformed about what the patient’s death will be like, or feel hostility from care staff about the prospect of withdrawing care. In some jurisdictions, lengthy court proceedings may be required. As described above, cultural differences may also influence a family’s willingness to withhold or withdraw life‐sustaining treatment, as well as other factors like education, religious beliefs, gender, and income.35
Levin, op. cit*.* note 32; Van Keer, R. L., Deschepper, R., Francke, A. L., Huyghens, L., & Bilsen, J. (2015). Conflicts between healthcare professionals and families of a multi‐ethnic patient population during critical care: an ethnographic study. *Critical Care*, *19*, 441–456.10.1186/s13054-015-1158-4PMC469933826694072



Clinical teams that provide compassionate and respectful support for families, provide high‐quality information, and prepare families for what the dying process will be like, can reduce the negative psychological impact of this decision. Research suggests that high‐quality palliative care that is responsive to these issues can result in a ‘good death’ for the patient, and be ethically acceptable for the family.36
Kitzinger, J., & Kitzinger, C. (2018). Deaths after feeding‐tube withdrawal from patients in vegetative and minimally conscious states: A qualitative study of family experience. *Palliative Medicine*, *32*(7), 1180–1188.10.1177/0269216318766430PMC604173829569993
 However, particularly in certain cultural contexts, the best interests of the patient may be much more closely bound with the interests of their family, making a ‘good death’ for the patient more dependent on the family’s beliefs and preferences. Whether a patient’s life is worth living may be determined not only by their own values and interests, but those of their family as well.

Differences between the values of families and physicians can complicate decision‐making on behalf of patients, particularly in a multi‐ethnic care context.37
Van Keer, op. cit*.* note 35.
 The views of families regarding withdrawal of life‐sustaining treatment, for example, may be at odds with the prevailing ethos of the health system in which their family member is a patient. My primary focus to this point has been on cultural contexts in which the interests of the patient are the primary concern of physicians and surrogate decision‐makers. When life‐sustaining treatment is no longer a benefit to the patient, it should be withdrawn, even if this is emotionally difficult for families. At the same time, my argument allows for a plurality of views about what is in the best interests of a particular patient (i.e., what makes their lives worth living). Conversely, in contexts in which patient interests are secondary to other normative considerations, such as the sanctity of life or the interests of the family and community, my argument may be less persuasive (and would need to be supplemented by an argument for why a patient’s interests should be the primary concern of physicians and families).

### 9.3 The costs of aggressive treatment

A third, related objection is the potential for additional cost arising from aggressively treating a much higher proportion of severely brain injured patients. Because of the unpredictable nature of recovery after brain injury, and the uncertainty of diagnosing prolonged disorders of consciousness, some surrogate decision‐makers request that patients continue to receive life‐sustaining treatment for months or years, in the hopes of a ‘miracle’ recovery. Many patients who sustain severe brain injury, especially those surviving traumatic brain injuries, are relatively young and otherwise healthy; these patients can survive for several years after their injury. Providing open‐ended care for patients with severe brain injury already places a considerable strain on limited healthcare resources. Increasing the number of these patients by routinely providing aggressive treatment may not be sustainable. Indeed, concern about the costs of long‐term care may be a motivating factor in physicians withholding treatment.

Yet failing to routinely provide aggressive care already leads to the premature deaths of patients who would have gone on to make a good recovery from their injuries. This represents a major cost that must not be ignored. A physician’s duty is to provide the treatment that is in the best interests of the patient in front of them. Decisions to withhold or withdraw care at the level of individual patients should not be made on the basis of resource allocation concerns. Physicians should not be responsible for weighing the interests of their actual patients against the potential interests of other patients.

Research also suggests that aggressive care may be less costly than routine care, when accounting for the costs associated with long‐term nursing care and lost productivity. Whitemore and colleagues found that in the average 20 year‐old, aggressive care yields a cost of $1,264,000 (± $118,000), while routine care yields a cost of $1,361,000 (± $107,000) over the life of the patient.38
Whitemore, op. cit*.* note 18.
 Aggressive care remains significantly less costly than routine care until age 80. However, even if aggressive care in the elderly is more expensive than routine care, this may fall within the range of what society is willing to pay for treatment interventions. Aggressive treatment in patients over 80 years is estimated to cost society $88,507 per Quality Adjusted Life Year (QALY). This cost to society is much less than that of many other accepted interventions for older patients, such as many types of chemotherapy (which can exceed $100,000 per QALY).39
Ibid.



Cost‐effectiveness metrics like QALYs pose a challenge in this context, insofar as it may be difficult to evaluate a patient’s subjective quality of life. If we underestimate the value that patients with severe brain injury assign to their lives, we risk biasing the QALY calculation. This suggests that a comparison of aggressive treatment and non‐aggressive treatment after severe brain injury, measured in terms of expected QALYs, may be imprecise at an individual level. Nevertheless, it seems reasonable to interpret this evidence as providing at least preliminary support in favour of my proposal.

At the same time, the equitable distribution of scarce healthcare resources is a requirement of justice. The fact that a patient has been provided with life‐sustaining treatment does not in and of itself entitle them to the continuation of such treatment. If a patient is no longer benefitting from continued treatment, it should not be provided. The challenge of differentiating between patients with severe brain injury that may benefit from treatment and those that will not, underscores the need for continued research into generating accurate prognostic markers for recovery. For example, the continued development of functional neuroimaging research, to more accurately predict recovery in patients with disorders of consciousness, may be useful in making treatment allocation decisions.

## 10 CONCLUSION

Prognosis immediately after severe brain injury is highly uncertain. Yet many patients are allowed to die—through the withholding or withdrawal of life‐sustaining treatment—when there is a significant chance of a good recovery. I argue that when prognosis is uncertain, patients should be provided with aggressive care. However, if it becomes clear that a patient is no longer benefitting from continued treatment, they should be withdrawn from life‐sustaining treatment, including artificial nutrition and hydration, and be allowed to die.

The decision to withhold or withdraw life‐sustaining treatment from a patient is an incredibly difficult one, for both physicians and surrogate decision‐makers. Both parties want to do what they believe is best for the patient. Yet withholding or withdrawing treatment when prognosis remains highly uncertain risks killing a patient who would have recovered. While there may be costs to routinely providing aggressive treatment to severely brain injured patients, none are as costly as this.

